# Microbiota: An Emerging Biomarker in Colorectal Cancer

**DOI:** 10.3390/cancers13215530

**Published:** 2021-11-04

**Authors:** John Tsiaoussis, John Souglakos

**Affiliations:** 1 Department of Anatomy, Medical School, University of Crete, 70013 Heraklion, Greece; 2 Laboratory of Translational Oncology, Medical School, University of Crete, 70013 Heraklion, Greece; souglak@uoc.gr

This series of 10 articles (four original articles, five literature reviews, and one systematic review) is presented by international experts in the study of microbiota and its relation to colorectal cancer (CRC).

In recent years, the microbiota, especially in the intestinal microenvironent, has received increasing attention. Intestinal microbiota consists of numerous microbial species that collectively interact with the host, playing a crucial role in health and disease. CRC is the third most common cancer worldwide, posing a substantial humanistic and economic burden on patients, healthcare systems, and society. It is also characterized by a multifactorial etiology including genetics, lifestyle, and environmental factors. Intestinal microbiota may promote carcinogenesis, and alterations of its composition have been associated with CRC. Microbiota analysis requires DNA extraction followed by 16S rRNA gene-based profiling in a variety of samples including mucosal samples or scraping and fecal material. Since large sample sizes are needed for association studies between intestinal microbiota and CRC, established biorepositories, such as CRC biobanks, may be useful alternatives to prospective clinical studies, aiding the long-term storage of the biospecimens and high-quality reads [1].

Metagenomic analyses have demonstrated that the host microbiota mutually beneficial symbiosis existing under physiologic conditions is lost during a state of pathological microbial imbalance due to the alteration of microbiota composition (dysbiosis) and/or the genetic susceptibility of the host. Overlying gastrointestinal epithelial cells is a dynamic mucus layer, consisting of glycosylated proteins called mucins, that serves as an interface between luminal contents and epithelial cells and provides a habitat for commensal bacteria and signals to the underlying immune system. Aberrant mucin production and expression compromises the mucus layer and allows bacteria to come into close contact with the intestinal epithelium, potentially triggering unfavorable host responses and the subsequent development of CRC [2]. It has been shown that initiation of CRC development by intestinal microbiota requires the formation of polymicrobial biofilms in the inner colonic mucus layer. Biofilm results in the redistribution of colonic epithelial cell E-cadherin increases the permeability of the gut, and causes a loss of function of the intestinal barrier, all of which enhance intestinal dysbiosis. Hence, various strategies have been proposed to hinder colon carcinogenesis via targeting colon-associated biofilms [3]. CRC initiation is believed to result from the conversion of normal intestinal stem cells (ISCs) into cancer stem cells (CSCs). Unlike other stem cells elsewhere in the body, ISCs cohabit with the intestinal microbiota and mounting evidence suggests that there is significant crosstalk between host and microbiota at the ISC niche level, revealing a possible key role of microbiota dysbiosis in the aberrant reprogramming of CSCs in the initiation of CRC [4]. This evidence also implies that CRC evolves through the multiple acquisitions of well-established genetic and epigenetic alterations with an adenoma-carcinoma sequence progression. Specific pathogens could trigger these phenomena (e.g., *Escherichia coli* and enterotoxigenic *Bacteroides fragilis*) enhancing the colonization of the intestinal microenvironment by opportunistic oncogenic bacteria (e.g., *Fusobacterium nucleatum*) which further contribute to CRC progression. Hence, the analysis of intestinal microbiota composition in precancerous colonic lesions could have a predictive value in CRC, differentiating adenoma from non-adenoma patients, and therefore, preventing cancer progression. Nevertheless, the results of such correlations are still inconsistent and inconclusive in current literature and further research is required to elucidate the importance of such microbiota alterations [5]. Genetic susceptibility to CRC is also linked to microbiota dysbiosis. A study using a genetic mouse model of CRC (Winnie-APC^Min/+^) reveals that the onset of aberrant crypt foci (ACF) in genetically predisposed mice offspring may result from dysbiotic intestinal microbiota signatures of the breeders. In light of these results, preventive strategies developed to avoid dysbiosis could help to reduce the risk of tumor lesion onset and progression. These preventive approaches may be particularly effective during pregnancy and lactation to reduce a child’s risk of CRC development [6]. In all the above indicators of the involvement of microbiota dysbiosis in CRC carcinogenesis, one common characteristic is the induction of local or systemic inflammation caused by a plethora of inflammatory mediators secreted by both immune and tumor cells. The intestinal microbiota is closely linked to such mediators including tumor necrosis factor, nuclear factor kappa B, interleukins, and interferons. This association may be a potential therapeutic target, possibly through the application of fecal microbiota transplantation and/or probiotics in CRC patients [7].

Dysbiosis also leads to microbial translocation, which is the passage of microorganisms, their fragments, or their metabolites from the intestinal lumen into the blood circulation and other sites. Significantly higher rates of microbial fragments through blood PCR were observed in CRC patients that were also significantly associated with metastatic disease, shorter survival rates, and homozygous mutant alleles of TLR/VDR polymorphisms. Hence, the detection of microbial DNA fragments highlights the prognostic value of microbiota in CRC patients [8]. Another important aspect of intestinal microbiota is its interaction with therapeutic interventions of CRC. Iron deficiency anemia is a common complication of CRC and may require oral or intravenous iron therapy which could alter the colonic microbiota. It has been shown that the colonic tumor-associated (on-tumor) and paired non-tumor-associated adjacent (off-tumor) microbiota associated with intravenous iron-treated anemic CRC patients infers increased abundances of enzymes involved in iron sequestration and anti-inflammatory/oncogenic metabolite production, compared to oral iron-treated patients. This suggests that intravenous iron may be a more appropriate therapy to limit adverse microbial outcomes in CRC [9]. Surgery is the cornerstone of CRC treatment and the microbiota is significantly affected by colorectal surgery in combination with the various perioperative interventions, mainly mechanical bowel preparation and antibiotic prophylaxis. The altered postoperative composition of intestinal microbiota could be causative of severe complications including anastomotic leakage and surgical site infections. Importantly, the intestinal microbiota could also be utilized as a possible biomarker in predicting long-term outcomes after surgical CRC treatment [10].

All in all, this Special Issue of *Cancers* is a collection of articles discussing the involvement of microbiota in CRC, revealing its role as a novel biomarker for CRC carcinogenesis, prognosis, and treatment.
